# Lost to follow-up: reasons and characteristics of patients undergoing corneal transplantation at Tenwek Hospital in Kenya, East Africa

**DOI:** 10.11604/pamj.2020.36.95.19993

**Published:** 2020-06-15

**Authors:** Belinda Ijeoma Ikpoh, Allen Kunselman, Christy Stetter, Michael Chen

**Affiliations:** 1 Penn State College of Medicine, Hershey, Pennsylvania, United States of America,; 2 Department of Public Health Sciences, Penn State College of Medicine, Hershey, Pennsylvania, United States of America,; 3 Tenwek Hospital, Bomet, Bomet County, Kenya; 4 Penn State Eye Center, Hershey, Pennsylvania, United States of America

**Keywords:** Follow-up, appointment adherence, compliance, cornea, corneal transplantation, penetrating keratoplasty, Kenya, Africa

## Abstract

**Introduction:**

corneal transplantation is a surgical procedure requiring consistent long-term follow-up to maximize the chance of graft survival. The purpose of this study was to explore patient characteristics and reasons for being lost to follow-up (LTFU).

**Methods:**

a retrospective review of clinical records from January 2012 to October 2014 was conducted of patients who received corneal transplantation at Tenwek Hospital. At the time of chart review, all patients who provided a mobile phone number were contacted to answer a phone questionnaire. Logistic regression was used to assess the association of each patient characteristic, separately, with the outcome of LTFU.

**Results:**

of the 118 patients that met inclusion criteria, 40 (33.9%) were considered LTFU by failing to follow up at Tenwek Hospital to at least one year postoperatively. The odds of LTFU for patients age 60 and older were 3.78 times that of those who were 18-59 (95% CI: 1.21-11.80]; p-value=0.02). The odds of LTFU for patients with a preoperative diagnosis of pseudophakic bullous keratopathy were 3.83 times that of those with a preoperative diagnosis of keratoconus (95% CI: [1.13-12.94]; p-value=0.03). Education level, employment status, distance from the hospital, and possession of a mobile contact number appeared marginally associated with follow-up status, though not statistically significant at the 0.05 significance level. Financial barriers were the most commonly cited reason for LTFU (42.4%, n=14).

**Conclusion:**

certain reasons and patient characteristics may be associated with follow-up adherence. Identifying these factors may help providers identify patients who are at a higher risk of LTFU and influence providers in medical decision-making and system-based interventions when offering corneal transplantation.

## Introduction

Blindness and visual impairment result in morbidity and substantial declines in quality of life. Corneal disease is one of the leading causes, affecting an estimated 4.5 million people worldwide [1,2]. Functional vision in certain etiologies of corneal blindness can be successfully restored by corneal transplantation, of which full-thickness corneal transplantation (penetrating keratoplasty [PKP]) is the most commonly performed method worldwide [3].

Outcomes of PKP at Tenwek Hospital, a tertiary care mission hospital in a rural low-resource setting located approximately 240 kilometers (4 hours) west of Nairobi, were previously reported [4]. While this study reported a favorable rate of improvement in uncorrected visual acuity and a favorable one-year graft survival rate, a significant percentage of the patient population (33.9%) was lost to follow-up (LTFU) at one year. This was despite every patient providing a verbal commitment to follow up at Tenwek Hospital for at least one year, after which patients were given the option to seek future follow-up care with an outside provider if they chose to do so.

Long-term follow-up is necessary to maximize the chance of graft survival, given the potential postoperative complications of PKP, such as graft rejection, traumatic dehiscence, increased intraocular pressure leading to glaucoma, infectious keratitis and suture-related complications [5]. Maximizing the chance of graft survival is important, not only because the risk for graft failure increases with each subsequent transplant [6], but also because of the global shortage of donor corneas, with an estimated single donor for every 70 recipients worldwide [3].

Therefore, particularly in low-resource settings, it is important for providers who offer corneal transplantation to identify and mitigate patient factors associated with LTFU, in order to help determine the best allocation of donor tissue and to maximize the lifespan of the corneas transplanted. As the World Health Organization (WHO) and the International Agency for the Prevention of Blindness created a global initiative to eliminate avoidable blindness by 2020, it is important to target patients at risk for being LTFU and provide them with appropriate interventions and pragmatic initial treatment options [7]. The aim of this study was to explore the reasons and patient characteristics associated with LTFU among patients who underwent corneal transplantation at Tenwek Hospital in Kenya, East Africa.

## Methods

A retrospective analysis of clinical records was performed in October 2015 of patients who underwent primary PKP for optical purposes between January 2012 and October 2014. LTFU was defined as permanently ceasing to appear for examination at Tenwek Hospital prior to one year postoperatively, or ceasing to appear for examination prior to one year postoperatively and reappearing after postoperative month 18.

At the time of chart review, all patients were called on their mobile phones to answer a questionnaire regarding additional demographic details, including education level, occupation level, place of residence and time of one-way travel to the hospital. Patients who were considered LTFU were additionally asked for the primary reason they failed to follow up. Occupational level was classified according to the International Standard Classification of Occupations (ISCO) [8]. Patients age 17 years or younger were excluded from analysis involving education level and occupation, because it was presumed that persons in this age group did not yet have the opportunity to complete a university level education or have formal employment. Median travel time from Tenwek hospital was four hours. Median transport fare was 600 Kenyan shillings (approximately $5.92 USD) [9].

Logistic regression was used to assess the association of each patient characteristic (i.e. demographic, visual acuity and surgical satisfaction) separately, with the outcome of LTFU. The effect size for each association was quantified via the odds ratio and 95% confidence interval.

The design of this study was reviewed and approved through the Tenwek Hospital institutional review and ethics committee and the Penn State College of Medicine institutional review board. The requirement for informed consent was waived. All data was fully anonymized before access by the researchers.

## Results

**Figure 1:**
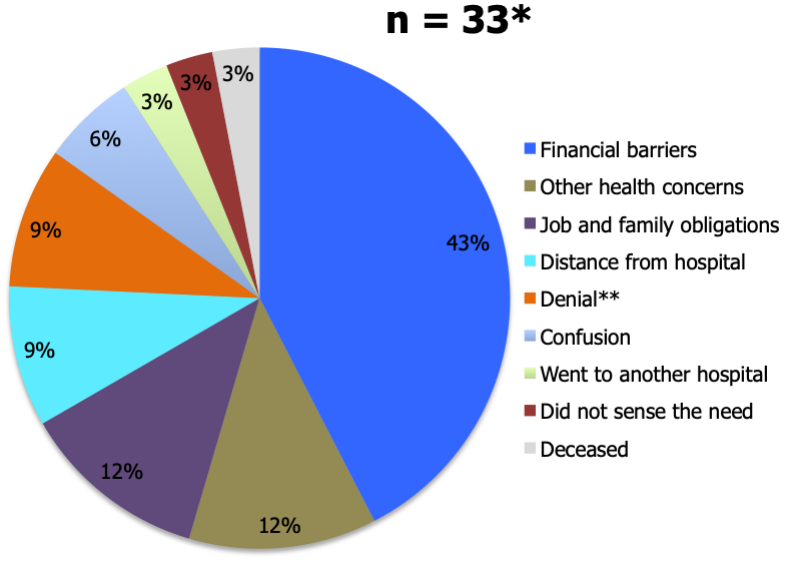
reasons for loss to follow-up

Between January 2012 and October 2014, 118 PKP patients met inclusion criteria for this study. The range of follow-up for this sample population was zero to 40 months, with an average duration of 19 months. Forty patients (33.9%) were LTFU. The primary reasons for being LTFU are depicted in Figure 1. Notably, 42.4% of the patients cited financial barriers as their main reason for being LTFU.

Associations of patient demographic, visual acuity and surgical satisfaction information with LTFU are shown in Table 1. Notably, the odds of LTFU for patients age 60 and older were 3.78 times that of those who were age 18-59 (95% CI: [1.21-11.80]; p-value=0.02). The odds of LTFU for patients with a preoperative diagnosis of pseudophakic bullous keratopathy (PBK) were 3.83 times that of those with a preoperative diagnosis of keratoconus (95% CI: [1.13-12.94]; p-value=0.03). In an attempt to fit a multivariable logistic regression model including both patient age and preoperative diagnosis as independent factors, as well as with and without their interaction, these factors were not statistically significant at the 0.05 level. Education level, employment status, distance from the hospital and possession of a mobile contact number appeared marginally associated with follow-up status, though not statistically significant at the 0.05 significance level.

**Table 1:** patient characteristics associated with loss to follow-up

Category	Characteristic	LTFU/Total Patients (LTFU %)	Odds Ratio (95% CI)	P-value
Age	60+	9/16 (56.3%)	3.78 (1.21, 11.80)	0.02
	<18	15/39 (38.5%)	1.84 (0.78, 4.33)	0.17
	18-59	16/63 (25.4%)	Ref	
Gender	Female	14/41 (34.1%)	1.02 (0.46, 2.26)	0.97
	Male	26/77 (33.8%)	Ref	
Hours from hospital*	>4	18/44 (40.9%)	2.20 (0.89, 5.45)	0.09
	≥4	11/46 (23.9%)	Ref	
Transport Fare*	>600 Kenyan shillings	15/39 (38.5%)	1.56 (0.62, 3.96)	0.35
	≥600 Kenyan shillings	12/42 (28.6%)	Ref	
Preoperative Diagnosis**	Pseudophakic Bullous Keratopathy	8/13 (61.5%)	3.83 (1.13, 12.94)	0.03
	Corneal scar	8/26 (30.8%)	1.06 (0.41, 2.79)	0.90
	Keratoconus	23/78 (29.5%)	Ref	
Preoperative visual acuity of contralateral eye	<6/18	27/72 (37.5%)	1.52 (0.68, 3.39)	0.30
	≤6/18	13/46 (28.3%)	Ref	
Nairobi Resident	No	30/91 (33.0%)	1.28 (0.42, 3.92)	0.67
	Yes	5/18 (27.8%)	Ref	
Education Level***	Other	12/30 (40.0%)	2.58 (0.89, 7.51)	0.08
	Completed college or university education	8/39 (20.5%)	Ref	
Occupation Level***	Unemployed	6/12 (50.0%)	3.14 (0.87, 11.32)	0.08
	Student or employed	14/58 (24.1%)	Ref	
Adverse Events	No	24/70 (34.3%)	1.04 (0.48, 2.27)	0.92
	Yes	16/48 (33.3%)	Ref	
Mobile Contact Number	No	5/8 (62.5%)	3.57 (0.81, 15.79)	0.09
	Yes	35/110 (31.8%)	Ref	
Satisfaction with Surgery	No/unsure/refused to answer	6/14 (42.9%)	1.83 (0.58, 5.79)	0.30
	Yes	27/93 (29.0%)	Ref	

CI = confidence interval. Ref = reference group. P-value < 0.05 is statistically significant. * Categories are based on the median; Nairobi residents excluded ** One patient with diagnosis of chemical injury was excluded *** Patients 17 and younger were excluded

## Discussion

This paper reports the follow-up rates of patients undergoing PKP at Tenwek Hospital in rural Kenya, and to our knowledge is the first paper to specifically address corneal transplantation follow-up rates involving any country on the African continent. Reasons identified by the patients in this study for being LTFU included financial barriers, confusion surrounding follow-up instructions and not sensing the need to return, all of which appear to align with the WHO reported risk factor categories associated with patient non-adherence [10].

Given our data, patient age and preoperative diagnosis may be providing similar information (i.e. collinear) with respect to LTFU, which prompted the use of multivariable logistic regression model. Both patient age and preoperative diagnosis as independent factors, as well as with and without their interaction, were not statistically significant at the 0.05 level. This is likely because in the previously reported data, 11 of the 13 (84.6%) patients diagnosed with PBK were over 60 years of age; whereas, only 4 of the 26 (15.4%) patients diagnosed with corneal scar and 1 of the 78 (1.3%) patients diagnosed with keratoconus were over 60 years of age [4].

Financial barriers were the most commonly cited reason for being LTFU (42.4%), which could be due to a variety of reasons in a country where the gross domestic product per capita in 2015 was approximately 137,724 Kenyan shillings ($1,337 USD) [11]. A patient undergoing PKP must not only be able to make follow-up appointments and have access to skilled ophthalmologists postoperatively, but also be able to obtain and administer steroid eye drops to avoid graft rejection [12]. While the donor tissue used for the corneal transplants in this study were donated and the costs of surgery were subsidized, patients were charged a nominal fee at the time of surgery. However, this did not cover the fees of the postoperative follow-up clinic visits, which over the course of the study cost between $1 to $2 USD per visit, and a typical patient was expected to return to clinic approximately six to eight times in the first year. The surgical fee also did not cover the price of the steroid eye drops, which cost approximately $2 USD per month supply. Creating a bundled fee to include essential components of postoperative care, such as eye drops and follow-up visits, may increase follow-up adherence, since financial allocations would have already been made to fund this portion of the treatment. Alternatively, being transparent about anticipated follow-up fees and other postoperative costs prior to surgery, which will give patients the opportunity to assess their financial obligations and help predict their ability to pay in the future, is an additional way of preemptively addressing financial issues that may arise.

The WHO framework on social determinants of health proposed that education captures the transition to socioeconomic position and is also a strong predictor of future employment and income [13]. Though not statistically significant at the 0.05 significance level, our study suggests that lower education level and lack of employment are possibly associated with LTFU. The association of LTFU with these social determinants of health is supported by previous reports in the literature regarding certain populations of patients undergoing eye surgery. In the only other report in the literature regarding follow-up in a cornea transplant population, Crawford *et al*. reported that PKP patients in New Zealand treated at private centers were more likely to attend appointments compared to those treated at public facilities [14]. If we associate treatment at private versus public centers as a reflection of financial security as the authors had proposed, our study is substantiated by the notion that financial standing is associated with LTFU. In relation to other eye surgeries, a study in rural China on patients who received cataract surgery found statistical significance between follow-up adherence and higher income [15], and a separate study in rural China investigating follow-up on patients who underwent trabeculectomy surgery found poor follow-up to be associated with elementary or less education and lower family annual income [16]. Our study suggests that patients who traveled more than four hours one way to and from the hospital may be more likely to be LTFU. This at first appears to be the opposite of the findings reported by Crawford *et al*. where higher rates of appointment compliance were inversely associated with proximity to the treatment center [14]. However, this may be because it is the responsibility of New Zealand district health boards to provide funding for patients within their districts and travel expenses are covered if patients are required to travel outside of their district to receive health services [14]. Thus, both their study and this present study support the notion that removing financial barriers may result in increased levels of appointment adherence. In general, subsidized care at distant facilities may give patients the false perception that it will be less costly to undergo the surgical procedure at the distant facility. However, as our study suggests that distance is a barrier to follow-up care, in the situation where the distant facility may charge less than a local facility when providing comparable services, patients should be mindful to factor in the costs of transportation and other associated costs of travel.

Of the 40 patients (33.9%) in this study LTFU, five of these patients (12.5%) did not have a mobile contact number. In contrast, only three (3.8%) of the 78 patients who were not LTFU did not have a mobile contact number. When the phone surveys from this study were conducted in 2015, mobile phone penetration in Kenya was 88% [17]. Our study suggests that, when it comes to stratifying risk of LTFU, providers should be cognizant of the patient´s mobile phone status, as lack of a mobile contact number may indicate a higher probability of LTFU.

According to the Communications Authority of Kenya, Kenya has an estimated mobile phone penetrance of 106.8% as of March 2019 [18]. There may be promise with the use of mobile health (mHealth) services to increase follow-up appointment adherence. According to the WHO, mHealth is the use of mobile and wireless technologies to support the achievement of health objectives [19]. Kenya has been selected as a pilot site for mHealth initiatives in the past, such as *Helping Babies Breathe* and the use of short messaging services to increase return rates for immunization services [20]. Applying the use of mHealth-related services to surgical eye care, a study in China that focused on the effect of WeChat, a smartphone messaging app, on follow-up adherence of pediatric patients, found attendance rates in the WeChat group to be significantly higher than the control group with respect to total follow-up attendance [21]. Advances with mHealth or other mobile technology could facilitate communication and decrease LTFU rates.

Limitations of this study include the relatively small study population size. The relatively small number of patients in this study accurately reflects how rarely corneal transplantation is performed in Kenya, as Tenwek Hospital, over the study period, was one of the leading providers of corneal transplantation in Kenya. The large LTFU rate captures the challenging nature of performing corneal transplantation on this particular patient population, as LTFU occurred despite every patient providing verbal commitment to follow up at Tenwek Hospital for at least one year. It is unlikely LTFU was due to poor postoperative recovery or loss of confidence to return, since the vast majority of those who were LTFU expressed satisfaction with the surgical procedure on the phone questionnaire. This study suggests that future prospective studies on larger populations of PKP patients may be beneficial to determine if the implementation of the proposed suggestions from this study, such as bundled packages and messaging reminders, will have a significant effect on follow-up outcomes.

## Conclusion

Especially in situations where the supply of donor corneas is limited, it is important to identify PKP patients likely to be LTFU, in order to help determine the best allocation of donor tissue and to maximize the lifespan of the corneas transplanted. Elucidating the reasons and patient characteristics associated with a higher risk of LTFU may aid providers in medical decision-making and influence health systems in system-based interventions when offering corneal transplantation.

### What is known about this topic

- Corneal transplantation requires adequate follow-up to maximize graft survival;
- LTFU was associated with ethnicity and socioeconomic factors based on a New Zealand study of PKP patients.

### What this study adds

- Financial reasons may be a significant reason for LTFU in corneal transplantation patients in rural Kenya;
- Age and preoperative diagnosis are collinearly associated with LTFU;
- Education level, employment status, distance from the hospital and possession of a mobile contact number appear marginally associated with LTFU.
